# A quantitative reverse transcription-polymerase chain reaction for detection of Getah virus

**DOI:** 10.1038/s41598-018-36043-6

**Published:** 2018-12-05

**Authors:** Sing-Sin Sam, Boon-Teong Teoh, Cheah-Mun Chee, Noor-Adila Mohamed-Romai-Noor, Juraina Abd-Jamil, Shih-Keng Loong, Chee-Sieng Khor, Kim-Kee Tan, Sazaly AbuBakar

**Affiliations:** 1 Tropical Infectious Diseases Research and Education Centre (TIDREC), University of Malaya, Kuala Lumpur, Malaysia; 2 Department of Medical Microbiology, Faculty of Medicine, University of Malaya, Kuala Lumpur, Malaysia

## Abstract

Getah virus (GETV), a mosquito-borne alphavirus, is an emerging animal pathogen causing outbreaks among racehorses and pigs. Early detection of the GETV infection is essential for timely implementation of disease prevention and control interventions. Thus, a rapid and accurate nucleic acid detection method for GETV is highly needed. Here, two TaqMan minor groove binding (MGB) probe-based quantitative reverse transcription-polymerase chain reaction (qRT-PCR) assays were developed. The qRT-PCR primers and TaqMan MGB probe were designed based on the conserved region of nsP1 and nsP2 genes of 23 GETV genome sequences retrieved from GenBank. Only the qRT-PCR assay using nsP2-specific primers and probe detected all two Malaysia GETV strains (MM2021 and B254) without cross-reacting with other closely related arboviruses. The qRT-PCR assay detected as few as 10 copies of GETV RNA, but its detection limit at the 95% probability level was 63.25 GETV genome copies (probit analysis, *P* ≤ 0.05). Further validation of the qRT-PCR assay using 16 spiked simulated clinical specimens showed 100% for both sensitivity and specificity. In conclusion, the qRT-PCR assay developed in this study is useful for rapid, sensitive and specific detection and quantification of GETV.

## Introduction

Getah virus (GETV) is mosquito-borne virus belonging to the genus *Alphavirus* of the family *Togaviridae*. It is grouped under the Semliki Forest virus complex, together with other Old World Alphaviruses including Chikungunya virus (CHIKV), Ross River virus (RRV), O’ Nyong-Nyong virus (ONNV), Berbaru virus and Sindbis virus (SINV). GETV was first discovered in Malaysia in 1955, from *Culex gelidus* captured in a rubber plantation^1^. It is currently widely distributed across Northeast Asia and Australasia countries including Japan, Korea, China, Mongolia, Russia, Philippines, India, Cambodia, Vietnam, Thailand, and Australia^2–8^. GETV is transmitted primarily by *Culex* and *Aedes* mosquitoes. Serologic evidence of GETV infection has been reported in various species of vertebrates including human^9–11^.

GETV has emerged as an important equine pathogen causing outbreaks among the economically important racehorses in Japan and India^12–16^. The infected racehorses developed febrile illness, anorexia, hind limb edema, stiff gait, urticarial and swelling of submandibular lymph nodes^16^. The first outbreak occurred in Japan in year 1978–1983, affecting hundreds of racehorses at the Miho Training Center and Sakai Training Center^14,16,17^. In 1990, GETV infection outbreak was reported among the thoroughbred horses in India^15^. Recently in year 2014 and 2015, recurrent outbreaks of GETV infection occurred at the Miho Training Center in Japan despite of the use of GETV vaccine^18–20^. Further investigation revealed that the recurrent outbreaks were associated with an epizootic of GETV infection among pigs in the pig farms nearby the Miho Training Center. GETV caused fetal death and reproductive failure in pigs^21^. Recently in 2017, the first GETV outbreak among pigs occurred in China resulting in hundreds deaths in the piglets^22^.

Both horses and pigs act as an amplifying host of GETV as they developed high enough viremia titer to infect biting mosquitoes and maintain the GETV transmission cycle^18,23–25^. Active periodic GETV surveillance in these animals as well as mosquitoes is needed to monitor disease transmission and to prevent potential outbreaks. Having a right tool for rapid and specific detection of GETV infection, thus, is very important. Virus isolation and serological tests, for instance serum neutralization test, are commonly performed for the diagnosis of GETV infection^17,22,26^. However, these methods are laborious and time-consuming. Furthermore, the serological methods can be challenging due to the possible cross-reactivity between other alphaviruses. Various reverse-transcription polymerase chain reaction (RT-PCR) assay have been developed and used for detection of GETV RNA genome^27–29^. However, an assay for both detection and quantification of GETV is still limited in the present. Hence, in this study, we intended to develop a quantitative RT-PCR (qRT-PCR) assay for rapid detection and quantification of GETV.

## Results

### Design of GETV-specific qRT-PCR assay primers and TaqMan probe

**Figure 1:**
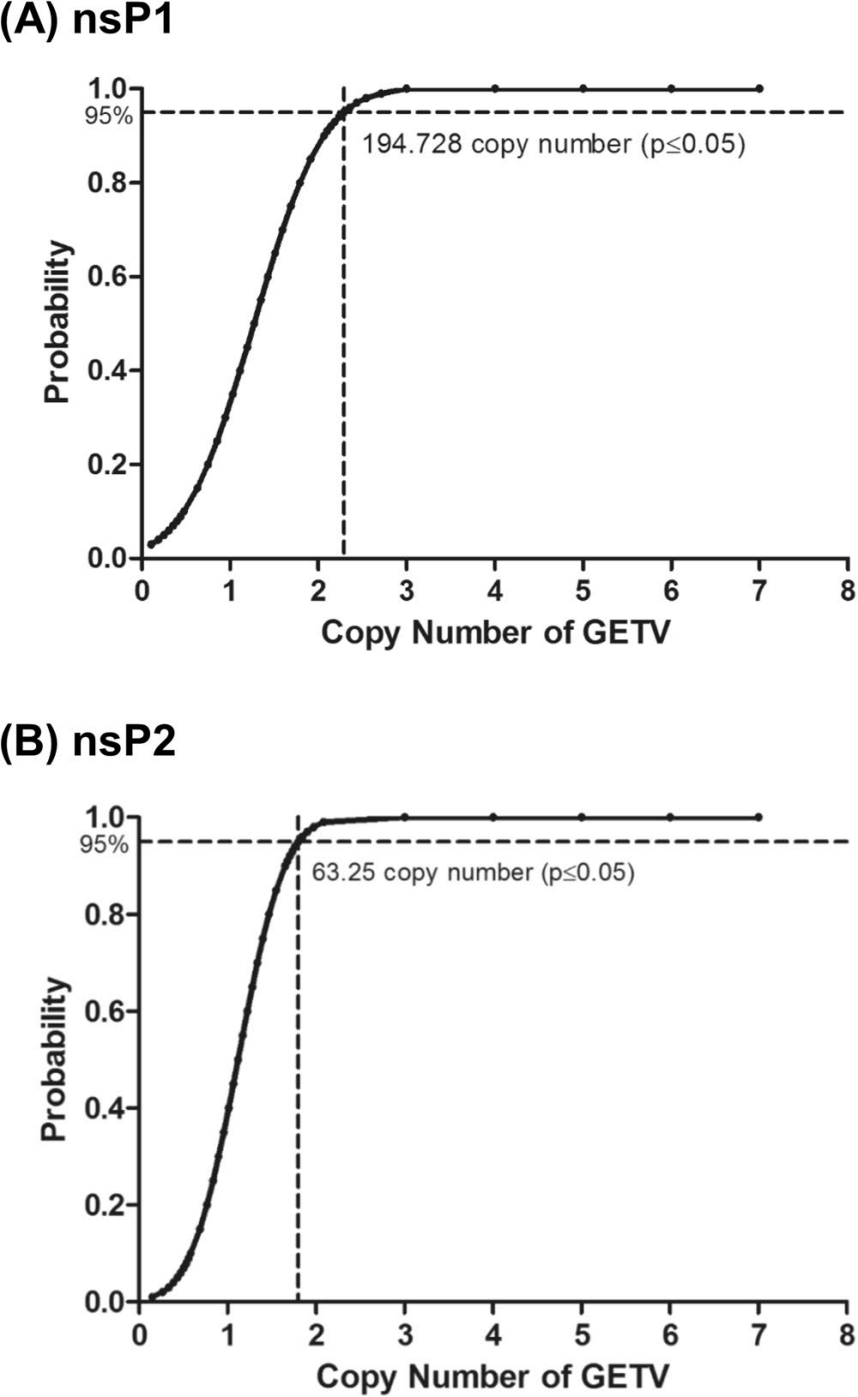
Detection limit of the GETV qRT-PCR assay using (**A**) GETV nsP1 and (**B**) nsP2 gene primers and probes. The probit regression curves were obtained from replicates of GETV RNA in 8 dilutions (10^7^, 10^6^, 10^5^, 10^4^, 10^3^, 10^2^, 50, and 10 copy numbers).

Two sets of GETV-specific primers and TaqMan minor groove binder (MGB) probes were designed based on the alignment of the conserved nsP1 and nsP2 gene region of 23 global GETV genome, respectively (Table 1 and Fig. 1). Both sets of qRT-PCR primer and probes detected the two GETV strains (MM2021 and B254) used in this study (Supplementary Fig. S1). The *in silico* study revealed that both sets of GETV primers and probes showed mismatches with sequences of other alphaviruses including Sindbis virus, Ndumu virus, Chinkungunya virus, O’Nyong Nyong virus, Semliki Forest virus, Una virus, Mayaro virus, Ross River virus, Middelburg virus, Barmah Forest virus, Eastern equine encephalitis virus, Western equine encephalitis virus and Venezuelan equine encephalitis virus (Supplementary Fig. S2).

**Table 1:** GETV qRT-PCR primers and TaqMan MGB probe used in this study.

Target Gene	Primers and probe	Sequence (5′ → 3′)
nsP1	F_459	GGAATCCCCGACTTTTTGC
	R_526	GGTACACGGCGACCTCAG
	P_484	^a^FAM-ACTGACGAGACGTGCC-MGB/NFQ
nsP2	F_2775	GCAACTGCAAATCGACTATCGT
	R_2838	TGTCAGACCCTGGGAAGCA
	P_2801	^a^FAM-ACGAGGTGATGACCGC-MGB/NFQ

^a^FAM, TaqMan fluorescent dye 6-carboxyfluorescein; MGB/NFQ, minor groove binder/non-fluorescent quencher. The nucleotide positions refer to the published complete genome of GETV (GenBank accession number: NC_006558).

### Detection limit of qRT-PCR assay

**Figure 2:**
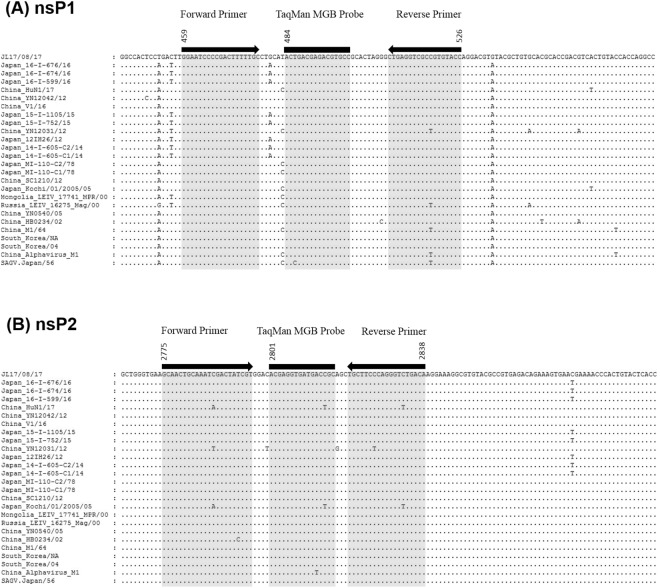
Map of qRT-PCR primers and probes in alignment with (**A**) the GETV nsP1 and (**B**) nsP2 sequences. The nucleotide positions refer to the published complete genome of GETV (GenBank accession number: NC_006558).

The detection limit of the qRT-PCR assay was determined by repeated independent testing on a panel of serially diluted viral RNA standard ranging from 10^7^ to 10^1^ copies. Both assays using GETV nsP1 and nsP2 primers and probes detected up to 10 copies of the RNA standard with 40% positivity, respectively. Using the GETV nsP1 primers and probes, the positive detection by qRT-PCR assay for the GETV RNA standard of 10^7^, 10^6^, 10^5^, 10^4^, 10^3^, 10^2^, 50, and 10 copies were 100% (1 of 1), 100% (5 of 5), 100% (5 of 5), 100% (5 of 5), 100% (5 of 5), 100% (5 of 5), 50% (2 of 4) and 40% (2 of 5), respectively. The detection limit of the qRT-PCR assay was 194.73 GETV genome copies at the 95% probability level (probit analysis, *P* ≤ 0.05) (Fig. 2). Using the GETV nsP2 primers and probe, the positive detection by qRT-PCR assay for the GETV RNA standard of 10^7^, 10^6^, 10^5^, 10^4^, 10^3^, 10^2^, 50, and 10 copy numbers were 100% (1 of 1), 100% (11 of 11), 100% (11 of 11), 100% (11 of 11), 100% (11 of 11), 100% (11 of 11), 90% (9 of 10) and 40% (4 of 10), respectively. The detection limit of the qRT-PCR assay was 63.25 GETV genome copies at the 95% probability level (probit analysis, *P* ≤ 0.05) (Fig. 2).

### Cross-reactivity of qRT-PCR assay

The cross-reactivity of GETV qRT-PCR assay was tested using RNA of the other closely related arboviruses common in the region, including CHIKV, SINV, dengue virus type 1 to 4 (DENV-1, DENV-2, DENV-3, and DENV-4), Asian Zika virus (ZIKV) P6-740, African ZIKV MR766, Japanese encephalitis virus (JEV), Langat virus (LGTV). The qRT-PCR using GETV nsP1 primers and probes showed cross-reactivity with CHIKV, SINV and DENV-1; the Ct values of the RNA samples were 35.56, 33.67, and 30.09, respectively (Supplementary Fig. S1). No cross-reactivity of the qRT-PCR assay using nsP2 primers and probe was observed with all the other alphaviruses or flaviviruses tested (Supplementary Fig. S1). Thus, only the qRT-PCR assay with nsP2 primers and probe was subjected to the subsequent evaluation study.

### Evaluation of qRT-PCR assay

The qRT-PCR assay using nsP2 primers and probe for detection of GETV RNA was evaluated by testing on a total of 16 simulated clinical samples containing GETV titers ranging from 1 × 10^5^ to 1 × 10^0^ plaque forming unit per ml (PFU/ml). The qRT-PCR assay detected up to as few as 1 PFU GETV in the simulated clinical samples. The qRT-PCR assay detected GETV RNA in all of the positive samples and none of the GETV negative samples showed positive amplification (Supplementary Table S1). The Diagnostic Test Calculator by Evidence-Based Medicine Toolbox calculated a sensitivity of 100% (95% CI = 78.5–100%) and specificity of 100% (95% CI = 34.2–100%) for the GETV qRT-PCR assay developed in this study (Table 2).

**Table 2:** Diagnostic performance of the qRT-PCR assay using the simulated clinical samples (*n* = 16).

	Results^a^	Simulated clinical samples		Sensitivity [% (95% CI^b^)]	Specificity [% (95% CI)]	PPV^c^ [% (95% CI)]	NPV^d^ [% (95% CI)]
		Pos [n (%)]	Neg [n (%)]				
qRT-PCR	Pos	14 (100.0)	0 (0.0)	100.0(78.5–100.0)	100.0(34.2–100.0)	100.0(78.5–100.0)	100.0(34.2–100.0)
	Neg	0 (0.0)	2 (100.0)				

^a^Pos, positive; Neg, negative.

^b^CI, confidence interval.

^c^PPV, positive predictive value.

^d^NPV, negative predictive value.

## Discussion

In the present study, two TaqMan MGB probe-based qRT-PCR assays targeting nsP1 and nsP2 genes of GETV were developed. The findings showed that the assay using nsP2 primers and probe was better for the detection and quantification of GETV RNA as it could detect all the GETV strains used in this study without cross-reacting with other common arboviruses including CHIKV, SINV, DENV-1, DENV-2, DENV-3, DENV-4, ZIKV P6-740, ZIKV MR766, JEV, and LGT. Probit analysis revealed that the detection limit of the qRT-PCR assay at the 95% probability level was 63 copies RNA. The qRT-PCR assay was sensitive and specific for the detection of GETV in all tested simulated clinical samples.

The qRT-PCR assay developed here used a TaqMan MGB probe, which gives an advantage of increased specificity and lesser background noise in comparison to the conventional TaqMan probe^30,31^. In this study, the GETV qRT-PCR primers and probe were designed based on the non-structural protein gene sequences due to their highly conserved nature in the genome yet contain enough genetic variations to differentiate from the other alphaviruses. Several previous studies have reported conventional single-plex and multiplex RT-PCR assays for detection of GETV RNA using primers targeting nsP1, capsid and E1 gene^27,29,32^. Recently, Shi and the group reported a TaqMan probe-based qRT-PCR assay for detection of GETV RNA^32^. The primers designed targeted conserved regions of nsP1 gene of GETV. However, the specificity of the primers was not tested against other closely related alphaviruses or flaviviruses but other livestock viruses. In this study, the GETV nsP1 primers and probe showed cross-reactivity with CHIKV, SINV and DENV, although the *in silico* study of the primers showed mismatches with the sequences of other alphaviruses. In contrary, the qRT-PCR assay using GETV nsP2-spesific primers and probe showed better sensitivity without cross-reacting with other closely related arboviruses that share a mosquito vector with GETV. Thus, this assay is specific for detecting GETV and important for virus surveillance in mosquitoes.

The qRT-PCR assays developed in this study detected up to as few as 10 copies of GETV RNA. The detection limit of the nsP1- and nsP2-specific primers and probes at the 95% probability level was determined to be 195 and 63 copies of viral RNA, respectively. Shi *et al*. has reported that the detection limit of their qRT-PCR assay was 25.5 copies RNA, which is at the same log scale as our assay using the nsP2-specific primers and probe. The information on the confidence level of the qRT-PCR assay of Shi *et al*., however, was not available.

In order to quantify GETV RNA in the samples, a panel of GETV RNA standard with known copy number is needed. Our GETV RNA standard were prepared using RNA *in vitro* transcribed from a plasmid vector carrying the nsP1 or nsP2 target sequences of GETV. The RNA copy number was calculated using formula based on the weight and sequence length of RNA transcript. In order to ensure the accuracy of RNA copy number, the RNA standard must be properly *in vitro*-transcribed. Linearization of the plasmid by single restriction enzyme digestion was performed before the *in vitro* transcription, this is to ensure that the RNA standard copies produced were all in the same length, which in turn enable the accurate calculation of the RNA copy number.

Considering the increasing GETV infection threats pose to animals, especially horses and pigs, leading to outbreaks in Japan and China^16,18,19,22^, a proper early detection tool in monitoring GETV activities in Malaysia is highly needed. In 1960s, the antibody against GETV has been frequently found in the horses and pigs in Peninsular Malaysia^11^ and yet to date the disease in the animals has not been reported. This could be due to the under-reporting and under-diagnosis because of unavailability of proper detection tools. Nevertheless, the following studies on GETV in the country are especially lacking. In Japan, GETV has been isolated from a range of animal samples including serum, saliva and nasal swab samples^23^. In this study, simulated clinical samples were used to assess the sensitivity and specificity of the qRT-PCR assay due to the lacking of well-characterized clinical samples of GETV in the region. The simulated clinical samples were prepared by spiking various concentrations of GETV into healthy human serum and saliva. The qRT-PCR developed in this study detected the GETV at titer of as few as one PFU/mL in both the simulated serum and saliva specimens. The findings showed that the qRT-PCR was sensitive enough to diagnose those infected animals which were still infectious. Early detection of the infected animal during the viremia phase allows immediate implementation of the disease control measures such as insecticide fogging and quarantine of the infected animals from subsequent mosquito bites, which in turn could reduce the transmission of the GETV. The discrepancy in the GETV RNA copies detected in serum and saliva samples, however, may indicate the differences in limit of detection of the qRT-PCR assay in different human matrices. Due to the small number of simulated clinical samples used in this study, the values for specificity, sensitivity, positive predictive value and negative predictive value need to be taken with caution. Further evaluation of the qRT-PCR in the field setting with a larger sample size of actual clinical samples and field-caught mosquitoes is warranted in future.

In conclusion, the qRT-PCR assay developed in this study is useful for rapid, sensitive and specific detection and quantification of GETV. The qRT-PCR assay would be useful not only for rapid diagnosis of GETV infection and virus surveillance but also for the research applications.

## Methods

### Getah viruses

Two Getah virus strains (MM2021 and B254) were used in this study. Strain MM2021 was provided by Dr. Robert Tesh (World Reference Center for Emerging Viruses and Arboviruses, The University of Texas Medical Branch, Galveston, USA). Strain B254 was isolated from *Aedes albopictus* in Malaysia in 2011–2014 (unpublished data). All the viruses were archived in the TIDREC Virology Laboratory repository. The viruses were propagated in C6/36 cells. All experiments in this study were performed in Biosafety Level-2 laboratory.

### Simulated clinical samples

The study obtained ethics approval from the UMMC Medical Research Ethics Committee (MREC ID No: 2017220-4938). All methods were carried out in accordance with relevant guidelines and regulations. All simulated clinical samples were prepared by spiking GETV into actual human saliva and serum obtained from a healthy volunteer with informed consent at final concentrations ranging from 1 × 10^5^ to 1 × 10^0^ PFU/ml. Serum and saliva spiked with serum free medium acted as negative samples.

### RNA extraction

Total RNA was extracted from 140 µl of infected cell culture supernatant or simulated clinical samples using QIAamp Viral RNA Mini Kit (Qiagen, Germany) following the manufacturer’s protocol. The RNA was eluted in 60 µl of nuclease-free water and stored at −80 °C until needed.

### Design of GETV-specific qRT-PCR assay primers and TaqMan probe

A total of 23 GETV complete genome sequences were retrieved from GenBank (Supplementary Table S2). Multiple sequence alignment was performed using Clustal X 2.0. The complete genomes of other alphaviruses were also included during the primer design. Two sets of GETV-specific primers and TaqMan MGB probes were designed based on the conserved regions of nsP1 and nsP2 genes, respectively, using Primer Express (Version 3.0.1) and GeneDoc (Version 2.7). The pairs of primers and probes were analysed based on the penalty points calculated by the Primer Express software. The GC content, melting temperature, presence of dimers and hairpin formation were further examined for each primer and probe using the IDT’s OligoAnalyzer Tool. The primers and probes were synthesized by Applied Biosystems™. The coverage of the qRT-PCR primers and probe was validated by evaluating the assay using viral RNA extracted from the different GETV strains.

### Preparation of GETV RNA standard

Two GETV RNA standards containing the nsP1 and nsP2 target sequences of GETV strain MM2021, respectively, were generated and amplified by RT-PCR using primer pairs designed using GeneDoc software (Supplementary Table S3). The RT-PCR reaction consist of 25 µL of 2x MyTaq One Step Mix, 0.5 µL of reverse transcriptase, 1.2 pmol of each forward primer and reverse primer, 1 µL of RiboSafe RNase inhibitor, 2 µL of RNA template and 19.5 µL of nuclease-free water. The RT-PCR was performed using Applied Biosystems Veriti Thermal Cycler according to the following condition: 20 min at 45 °C, 1 min at 95 C followed by 40 cycles of amplification (10 s at 95 °C, 10 s at 60 °C, 30 s at 72 °C). The nsP1 and nsP2 amplicons were ligated into two pGEM®-T Vectors respectively, using T4 DNA Ligase (Promega, USA) according to manufacturer’s protocol. The reaction was incubated for 1 hour at room temperature. The recombinant plasmids were linearized by restriction enzyme digestion using *Bam*HI (Promega, USA). The GETV RNA standards were *in vitro* transcribed from the linearized recombinant pGEM-T plasmids (Supplementary Fig. S3) using the MEGAscript^TM^ T7 transcription kit according to manufacturer’s protocol. The *in vitro* transcription was performed at 37 °C for at least 2 hr. DNase treatment and lithium chloride precipitation steps were also performed according to manufacturer’s protocol to purify the *in vitro* transcribed RNA. The purity and concentration of RNA was measured by spectrophotometer at 260 nm. The RNA copy number (copies/µL) was calculated based on the sequence length and weight of GETV RNA by using the web-based ENDMEMO DNA/RNA Copy Number Calculator (http://www.endmemo.com/bio/dnacopynum.php).

### Detection limit of qRT-PCR assay

The detection limit of the qRT-PCR assay using the primers and probes specific to GETV nsP1 and nsP2 genes was assessed using a panel of respective serially diluted *in vitro* transcribed GETV RNA standard (10^7^, 10^6^, 10^5^, 10^4^, 10^3^, 10^2^, 50, and 10 copy numbers). The detection limit test of qRT-PCR was repeated for at least four times for standard samples with less than 10^7^ RNA copies. The qRT-PCR was performed in a final volume of 15 μl containing 3.75 μl of 4× TaqMan Fast Virus 1-Step Master Mix, 0.75 μl of Custom TaqMan Gene Expression Assay (20X), 8.5 μl of nuclease-free water, and 2 µl of RNA template. Quantitative PCR measurement was performed using StepOnePlus real time PCR system (Applied Biosystems, USA) according to the following condition: 5 min at 50 °C, 20 s at 95 °C followed by 40 cycles of amplification (3 s at 95 °C, 30 s at 60 °C). Raw data was analyzed with StepOne Software v2.2.1 to determine copy number based on the threshold cycles (Ct). The Ct values above 35 were considered as negative. The efficiency of the qRT-PCR was measured from the slope of standard curve.

### Coverage and cross-reactivity of qRT-PCR assay

The coverage and cross-reactivity of the primers and probes were tested by evaluating the qRT-PCR assay on viral RNA extracted from two GETV strains (Malaysian strains: M2021 and B254), CHIKV, SINV, DENV-1, DENV-2, DENV-3, DENV-4, Asian ZIKV P6-740, African ZIKV MR766, JEV and LGTV. All of these viruses were obtained from the TIDREC viral repository.

### Evaluation of qRT-PCR assay

The sensitivity and specificity of the qRT-PCR assay for detection of GETV RNA was assessed using the simulated clinical samples as previously described. Serum and saliva samples were obtained from healthy volunteer and were spiked with a panel of 10-fold serial diluted GETV to make final concentrations ranged from 1 × 10^5^ to 1 × 10^0^ PFU/ml. In brief, 20 µL of 1 × 10^6^, 1 × 10^5^, 1 × 10^4^, 1 × 10^3^, 5 × 10^2^, 1 × 10^2^, 1 × 10^1^ PFU/ml GETV were added into 180 µl of serum or saliva samples. Serum sample and saliva sample spiked in with serum free media only acted as negative control.

### Statistical Analysis

All statistical analyses were performed using IBM SPSS Statistics, version 21 (IBM Corporation, New York, United States). A probit analysis was performed to calculate the detection limit of the qRT-PCR assay at a 95% probability level. The sensitivity and specificity of qRT-PCR assay were calculated using web-based EBM Diagnostic Test Calculator (http://ebm-tools.knowledgetranslation.net/calculator/diagnostic/).

## Electronic supplementary material

Supplementary Data
