# Supplementary material for: A quantitative reverse transcription-polymerase chain reaction for detection of Getah virus
Source: Sci Rep. 2018 Dec 5;8:17632. doi: 10.1038/s41598-018-36043-6 (PMC6281642; doi:10.1038/s41598-018-36043-6)
Supplement: Supplementary file 1 — Supplementary Data [file 41598_2018_36043_MOESM1_ESM.pdf]

Title: A quantitative reverse transcription-polymerase chain reaction for detection of Getah virus

Sing-Sin Sam[1], Boon-Teong Teoh[1], Cheah-Mun Chee[1], Noor-Adila Mohamed-Romai-Noor[1], Shih-Keng Loong[1], Chee-Sieng Khor[1], Juraina Abd-Jamil[1], Kim-Kee Tan[1] and Sazaly AbuBakar[1,2,*]

[1] Tropical Infectious Diseases Research and Education Centre (TIDREC), University of Malaya, Kuala Lumpur, Malaysia

[2] Department of Medical Microbiology, Faculty of Medicine, University of Malaya, Kuala Lumpur, Malaysia

*Corresponding author

Sazaly AbuBakar

Email: sazaly@um.edu.my

(A) nsP1

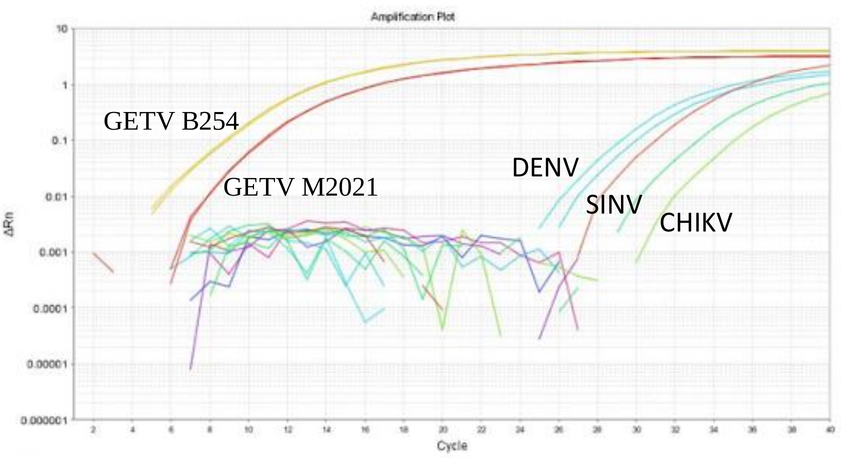

(B) nsP2

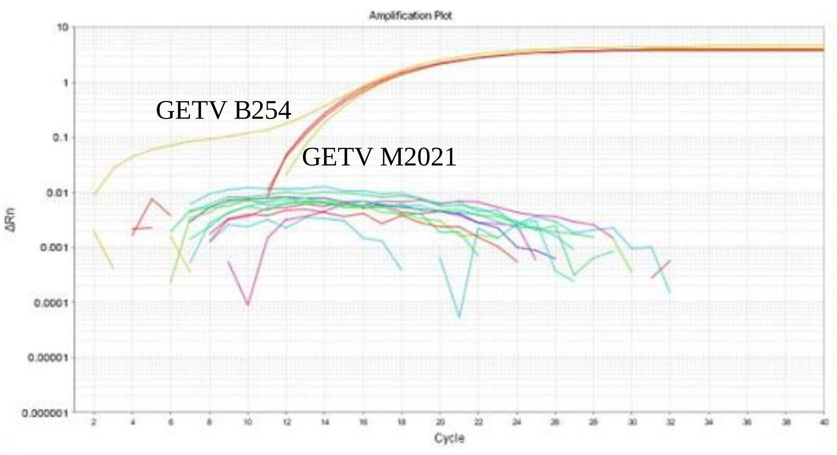

Figure S1. The coverage and cross-reactivity of the GETV qRT-PCR assay using (A) nsP1 and (B) nsP2 primers and probes. (A) and (B) The red line indicates amplification of GETV M2021 strain while the yellow lines indicates amplification of GETV B254 strain. (A) Amplification was observed against CHIKV, SINV and DENV. (B) No amplification was observed against other arboviruses.

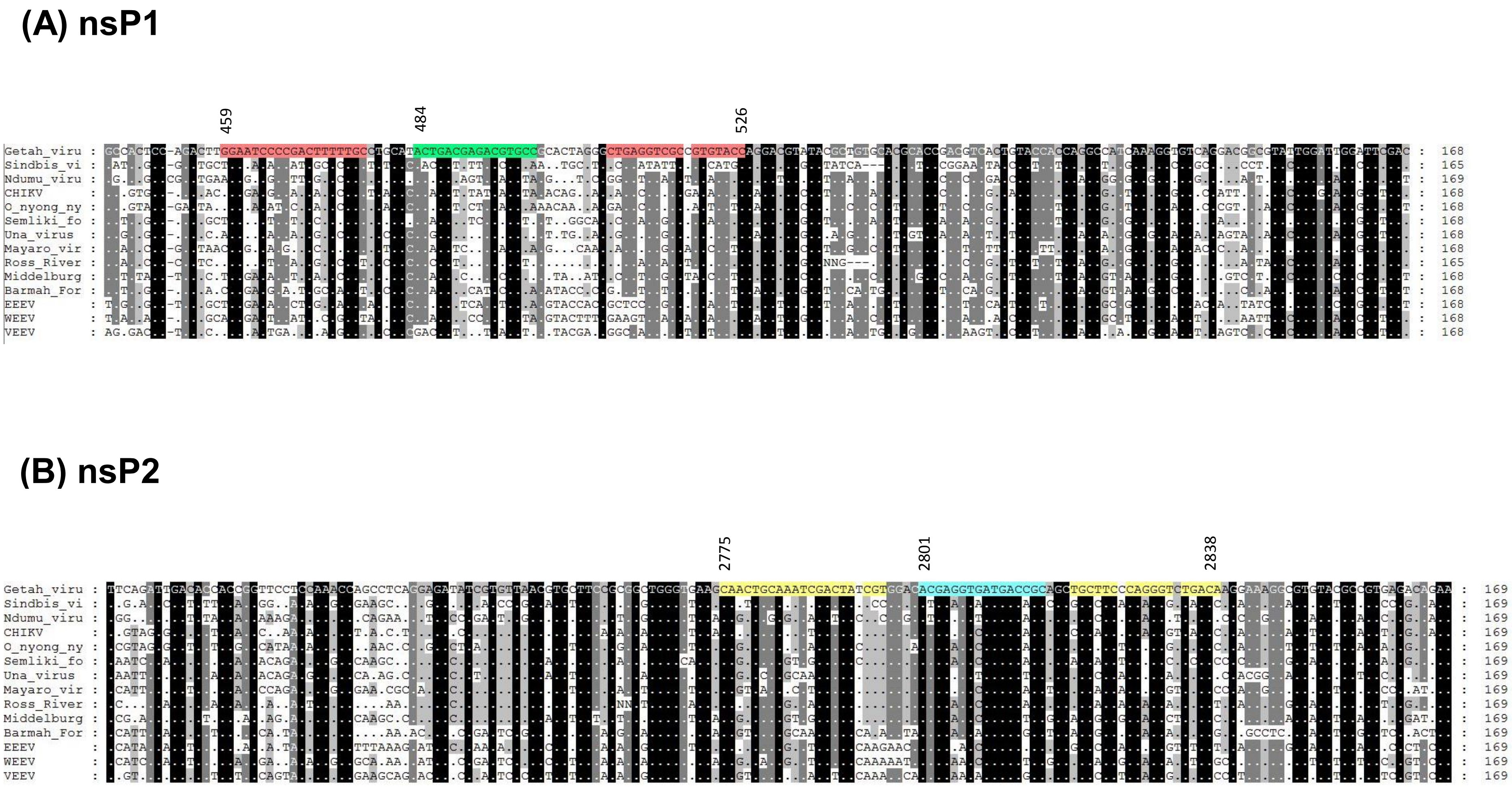

Figure S2. Map of GETV qRT-PCR primers and probes in alignment with (A) the nsP1 and (B) nsP2 gene sequences of other alphaviruses. The nucleotide positions refer to the published complete genome of GETV (GenBank accession number: NC_006558).

(A) nsP1

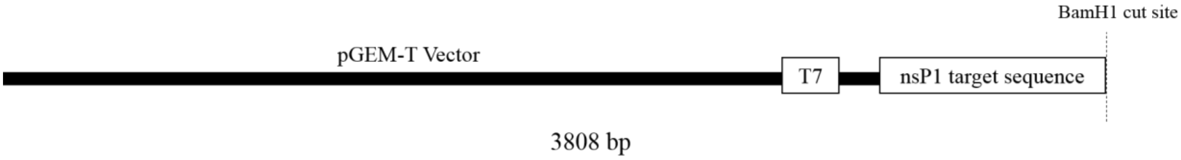

(B) nsP2

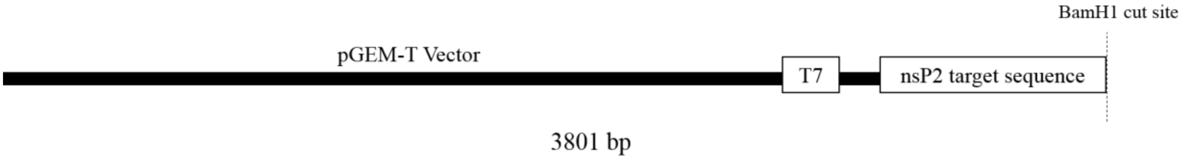

Figure S3. The map of the linearized recombinant pGEM-T Vector containing (A) the nsP1 and (B) nsP2 target sequence. T7, T7 promoter

Table S1. Quantification of GETV RNA copies in simulated clinical samples.

Virus titer of the simulated samples (pfu/ml)	Serum (mean RNA copy number/μL)	Saliva (mean RNA copy number/μL)
$1x10^5$	$9.04x10^6$	$9.12x10^6$
$1x10^4$	$3.93x10^5$	$1.09x10^6$
$1x10^3$	$5.30x10^4$	$1.46x10^5$
$1x10^2$	$5.76x10^3$	$2.96x10^4$
50	$6.80x10^2$	$4.72x10^3$
10	99.10	817.0
1	9.70	91.80
0	Undetermined	Undetermined

Table S2. List of GETV complete genome sequences retrieved from Genbank (n=23).

Accession No.	Strain	Country	Host	Year
AB859822	Kochi/01/2005	Kochi, Japan	Wild boar	2005
AB032553	Sagiyama virus	Japan	-	1999
AY702913	-	South Korea	Pig	2004
EF011023	M1	China	-	2006
EF631998	LEIV 16275 Mag	Russia	Mosquito	2007
EF631999	LEIV 17741 MPR	Mongolia	Mosquito	2007
EU015061	M1	Hainan, China	Mosquito	2007
EU015062	HB0234	Hebei, China	Mosquito	2007
EU015063	YN0540	Yunnan, China	Mosquito	2007
KY399029	GETV-V1	China	Pig	2016
KY434327	YN12031	Yunnan, China	Mosquito	2012
LC079086	MI-110-C1	Ibaraki, Japan	Horse	1978
LC079087	MI-110-C2	Ibaraki, Japan	Horse	1978
LC079088	14-I-605-C1	Ibaraki, Japan	Horse	2014
LC079089	14-I-605-C2	Ibaraki, Japan	Horse	2014
LC107870	SC1210	Sichuan, China	Mosquito	2012
LC152056	12IH26	Nagasaki, Japan	Mosquito	2012
LC212972	15-I-752	Ibaraki, Japan	Horse	2015
LC212973	15-I-1105	Ibaraki, Japan	Pig	2015
LC223130	16-I-599	Ibaraki, Japan	Horse	2016
LC223131	16-I-674	Ibaraki, Japan	Horse	2016
LC223132	16-I-676	Ibaraki, Japan	Horse	2016
NC_006558	-	South Korea	Pig	2004

Table S3. RT-PCR primers used for amplification of GETV nsP1 and nsP2 target sequences.

Primer	Sequence (5’-3’)	Product size
GETV_nsp1Fc	GAGCATTTTCGCATCTGGCTAC	808 bp
GETV_nsp1Rc	GGATCCGAATTCTGCGTGATGGGTAACTGC	
GETV_nsp2Fc	TGGATGCAAGAAAGGCGTAGAAAAC	801 bp
GETV_nsp2Rc	GGATCCGAATTCCTCTGAAAGCCAAGATGGTGTTC	
